# Active Home Literacy Environment: parents’ and teachers’ expectations of its influence on affective relationships at home, reading performance, and reading motivation in children aged 6 to 8 years

**DOI:** 10.3389/fpsyg.2023.1261662

**Published:** 2023-09-22

**Authors:** Marta Romero-González, Rocío Lavigne-Cerván, Sara Gamboa-Ternero, Gemma Rodríguez-Infante, Rocío Juárez-Ruiz de Mier, Juan Francisco Romero-Pérez

**Affiliations:** ^1^Department of Developmental and Educational Psychology, University of Malaga, Malaga, Spain; ^2^Centro de Neurociencias Infantojuvenil, Neuropsipe, Malaga, Spain

**Keywords:** home literacy environment, reading, motivation, affective relationships, primary education, program

## Abstract

Studies highlight the benefits of active Home Literacy Environment on learning and reading habits. This model is based on harnessing family involvement, resources and capabilities to create learning opportunities around reading, engaging in practices related to written language at home. However, it is less common to find applied research with children from the age of six, with older ages and already initiated in reading decoding. The aims are confirming and improving the expectations of families and teachers of a group of children (6–8 years old) regarding the effect of an active Home Literacy Environment program on the improvement of affective relationships between parents and children, reading performance, and children’s reading motivation. The method and procedure followed included carrying out an active Home Literacy Environment program for 18 months with a group of children (aged 6 to 8 years), their families and their teachers, and measures of all variables were collected at four times, using an *Ad Hoc* instrument designed for families and teachers. The results show that participants had high expectations about the influence of the Home Literacy Environment on the improvement of all variables even before the implementation of the program, improving their expectations about its effects on positive affective relationships at home and on reading achievement after the intervention. In conclusion, we suggest the need to continue investigating the effects of the active Home Literacy Environment program applied to children aged 6 to 8 years, older than those traditionally investigated. As well as their effects on family relationships, reading ability, and reading motivation.

## 1. Introduction

The fundamental role of families in children’s overall development and learning has been a focus of interest for many researchers over the years (De-La-Peña et al., 2018; among others). The first social and linguistic interactions of the child take place at home, with parents being primarily responsible for designing the right environment to foster learning opportunities, modeling desirable behavior for the young child, and providing the necessary scaffolding for building knowledge. However, the role of family in the acquisition of experiences and knowledge schemes continues throughout the child’s development, transforming the environment, transmitting a positive attitude toward learning, and generating expectations that influence the child’s motivation for certain tasks and activities.

At a later stage, activities carried out by teachers are added to the practices at home; teachers guide the child’s learning in the school context and advise families, guiding them how to enhance learning and, even more importantly, their motivation to learn at home. On the one hand, teachers set a series of guidelines to follow and provide recommendations to families through meetings and tutorials. On the other hand, they reinforce students’ achievements—individually or with family support—at home, encouraging children’s interest in impressing the teacher (and their classmates), obtaining their praise and rewards, and, consequently, enhancing children’s motivation for the task. Thus, a relationship is also established between the teacher and the student, associated with affective and emotional components, which requires the interest and involvement of the teacher and the student in the task (Ryan and Deci, 2017). A higher quality of these relationships is associated with higher motivation, and therefore, better academic performance, including reading performance (Roorda et al., 2017; Guay et al., 2019).

Furthermore, the influence of teachers continues beyond the school, as the child returns home and shares with his family what he has achieved, what comments the teacher has made, and how he feels, which also produces an increased sense of achievement and competence in the adults, leading them to maintain educational practices at home. Thus, directly, teachers influence their students’ motivation to learn—through their attitude and reinforcement at school—and, directly and indirectly they may have an impact on families’ interest in creating opportunities for increasing children’s motivation, through meetings and exchanges of information (directly or through the child, with documents such as academic notes, specific records, or the schedule).

### 1.1. Home literacy environment

Given the deep interest of psychology in general, and cognitive psychology in particular, in the influence of the family context on children’s learning, it is increasingly common to find studies and reports, both national and international, that analyze the predictive capacity of family variability in the acquisition and development of fundamental competencies and skills for the implementation of higher order processes such as reading (Ministerio de Educación y Formación Profesional, 2020; Vázquez-Cano et al., 2020, among others). Specifically, many researchers have focused on the study of Home Literacy Environment (HLE), defined as the quantity and quality of resources and skills that families possess for the creation of a context that facilitates learning opportunities, in which parents act as facilitators (Jiménez, 2012; Puglisi et al., 2017; Inoue et al., 2018; Wiescholek et al., 2018; among others); parents act as reading role models and encourage prolecting and reading activities, encouraging subsequent reading development and associated motivational aspects in children.

However, not all households are characterized as having an HLE, nor are all HLE the same and have the same effect on children’s reading knowledge and motivation. Burgess (2011) distinguishes two dimensions: (i) passive HLE, which refers to the parents’ skills and attitudes toward reading and their task as role models, and (ii) active HLE, which includes literacy activities carried out in the family in addition to the above. An active HLE has an apparently greater influence on the learning and development of reading, especially when formal literacy activities or *code-related interactions* are carried out, in which the child plays an active role and the task is focused on explicit learning of the written code, such as grapheme-phoneme conversion and reading comprehension strategies, as opposed to informal activities or *meaning-related interactions* that are associated with written language but do not directly manipulate it, an example of which is shared reading (Sénéchal and LeFevre, 2002, 2014). And, it is this active dimension that arouses the most interest in this study. On one hand, due to its flexible and dynamic nature, which allows adapting the model and its practices to the child’s individual characteristics (linguistic and reading level of the youngest, etc.), the family’s specificities (educational, linguistic, and reading levels of the adults in the household…), and their environment, in this case, the school context (socioeconomic and cultural context, the school’s interest in reading frequency and habits, improving the reading ability of its students, etc.). Not only that, but it also enables tailoring practices to meet the demands of reading tasks and stimulating both basic and complex cognitive processes, including fundamental motivational aspects that foster the child’s interest in reading.

### 1.2. Home literacy environment, reading and motivation

To understand the interaction between HLE and motivation, it is essential to delve deeper into the motivational aspects involved in children’s learning, and specifically, in reading. Only then will it be possible to understand the fundamental role of HLE in the improvement and enjoyment of reading.

#### 1.2.1. Reading as a process and behavior

Reading is a complex process that stretches beyond the academic realm as a beneficial skill for the development of brain structures and cognitive functions, as well as for activating emotions and feelings, providing insight into different ways of thinking and different perspectives, and so on (Oatley, 2016; Wolf, 2020). Reading involves perceiving and attending to stimuli, storing new knowledge, recovering and restructuring existing knowledge, anticipating and predicting, understanding the beliefs and desires of others, analyzing different points of view, analyzing the emotions and feelings of the characters and even the author, and so on. Therefore, its proper functioning requires the activation of multiple brain areas and participation of the processes, abilities, and functions that arise from these areas (Horowitz‐Kraus and Hutton, 2015), including the biological structures of the brain regions associated with affect and empathy, and the ability to understand mental states of others or theory of mind (Wolf, 2020).

Consequently, performing prolecting and reading tasks at home can help enhance these biological bases and positively influence psycholinguistic, cognitive, and behavioral processes and skills associated with reading or specific to it, as verified by this research team in previous studies, one of which has been published (Romero-González et al., 2021). In both studies, the positive effects of the application of an active HLE program on phonological awareness, vocabulary, comprehension of oral narratives, reading recognition, and speed and reading comprehension were corroborated in a group of participants aged 6 to 8 years, from a charter school in the city of Malaga (Spain).

The variables analyzed in relation to HLE have an impact beyond that on reading performance. Different studies confirm a direct correlation of both dimensions of HLE, whether studied jointly or separately, with frequency and reading habit from the age of 8 years, and even more importantly, with motivation and enjoyment of reading (Baker, 2013; Wirth et al., 2020).

The scope and impact of these findings can be observed not only at the national but also at the international level, through studies and reports that include representative samples from different countries and regions. These studies seek to determine the reading performance of children and adolescents, as well as the factors that influence literacy in this population. Traditionally, reports such as PISA measured the performance of representative samples of adolescents, aged 14–15 years, in specific reading tasks and made comparisons between: (i) the results obtained by different countries; (ii) the average scores of all participating countries, European and the OECD countries; (iii) the results of the regions that make up the same country, in the case of Spain, the autonomous communities; and (iv) the average scores of each autonomous community, the Spanish, European, and OECD averages. However, although the purpose of the test and the comparisons are maintained today, with an increase in research on learning and reading, the number of participants (including up to 70 countries in 2018) and the variables measured have also increased. Thus, the latest report pays special attention to the influence of contextual and motivational factors on reading ability, yielding data on the reading enjoyment index, which explains 10% of the variability in the reading performance of Spanish participants. In other words, children who enjoy reading the most read better. These findings have prompted education professionals to look for ways to encourage a taste for reading and incorporate new ways of increasing motivation for literature and for reading in their reading plans.

#### 1.2.2. Motivation as a construct

Motivation is a psychological process present in a large part of the activities and behaviors performed by human beings throughout the day in different contexts and situations. The nature of motivation and its effects on young children have led to numerous studies in recent years focused on determining the predictive validity of motivation to explain learning as measured through academic performance and success (2019; Regueiro et al., 2015; Guay and Bureau, 2018; Siegenthaler-Hierro et al., 2019; Rodríguez et al., 2020; among others). These attempts have prompted researchers from the fields of psychology, psycho-pedagogy, and education to not only accept the importance of this cognitive capacity in school results, but also in students’ persistence in studies and task completion (Pintrich, 2003; Guay et al., 2010; Guay and Bureau, 2018).

Classical authors such as Pintrich and De Groot (1990) distinguish several components associated with academic motivation: goal value, perceived competence, causal attributions, and emotional reactions. The desire for achieving personal goals, as well as the beliefs and value attached to them, drives students to conduct better time management in studies and be more consistent in homework completion (Linnenbrink and Pintrich, 2002; Regueiro et al., 2015; Rodríguez et al., 2020). All this favors students’ learning and academic performance, increasing their perception of competence and attributing successes to internal, stable, and controllable causes, which increases their sense of self-efficacy and task satisfaction. Specifically, according to the self-determination theory (Deci and Ryan, 1985, 2000; Ryan et al., 2008; Ryan and Deci, 2020), an increase in these components enhances the development of intrinsic motivation for academic tasks, defined as the internal tendency that leads individuals to act for the pleasure and enjoyment of the activity itself; that is, when the behavior is an end in itself and not a means to achieve another goal. Unlike extrinsic motivation, defined as an individual’s interest in performing a behavior to obtain a reward or avoid punishment, intrinsic motivation is promoted by internal factors, arousing great interest in the educational community, as it plays a crucial role in the increasingly complex behaviors that cause satisfaction and last over time.

Based on the understanding that motivation is a construct linked to multiple factors and composed of several elements, Guay et al. (2010) highlighted the need to study motivation in a broad manner in relation to school (including interest in learning, establishing relationships with the peer group, etc.), as well as in association with a specific subject or element (science, reading, etc.), that is, a specific domain (Green et al., 2007). Among these domains, in the last 20 years, interest in reading and the effect of motivation on reading are prominent not only in terms of reading performance but also the quality of reading (Schiefele et al., 2012, 2016), including reading behavior and habits.

#### 1.2.3. Motivation for reading: interaction with the home literacy environment

Siegenthaler-Hierro et al. (2019) and Ruiz et al. (2019) allude to the need to investigate the relationship between reading and motivation from an early age. In their works, belonging to the same project, they investigated the association between the learning motivation of 208 children, aged 5 to 6 years, as perceived by their teachers, and their reading ability (decoding of graphemes, lexical, syntactic and semantic processes) measured 2 years later. The results showed that the group of students with lower scores on motivation, especially on task persistence, also presented lower reading performance. This leads us to believe that increasing reading motivation will also mean fostering curiosity for the act of reading and establishing a reading habit among young students. For example, active HLE programs that would increase reading competence and autonomy, relationships and experiences with parents, positive memories of time spent reading with family, and so on, by increasing the child’s motivation to carry out activities associated with the written code with his or her family.

The act of reading is a demanding behavior that involves great cognitive effort not only during the first years of learning but throughout life. The current context in which the child and adolescent population is developing, characterized by the presence of increasingly accessible technology and its numerous forms of entertainment, can dissuade both children and adolescents from indulging in other activities, including reading. Therefore, the role of motivation and related brain structures, such as the limbic system, in reading is fundamental. Not only would it help the child choose reading over other activities but would also enhance the functioning of processes directly involved in the act of reading, such as memorization.

Specifically, among the brain structures related to motivation, the amygdala is a region that establishes connections with numerous structures in the brain. One of them is the septal area, responsible for modulating pleasant sensations and level of alertness; it is also connected with the hippocampus, the activation of which is very important during memorization. In view of this relationship and the mechanisms under which the aforementioned brain regions operate, researchers such as Almaguer-Melián and Bergado-Rosado (2002) The influence of emotions and motivation on the consolidation of memory and, therefore, on learning. A lesion in the septal area or stressful situations that alter the functioning of the amygdala can lead to changes in brain activity and in the connections that regulate the action of the hippocampus, and thereby, either enhance or impair the learning process. Thus, given the characteristics of reading and learning to read, as well as the role of motivation in this process, we believe it is essential to design programs for reading and learning that seek to improve motivational aspects of reading, such as active HLE programs (Siegenthaler-Hierro et al., 2019; Cho et al., 2021).

Nevo et al. (2020) conducted an investigation with 121 participants aged 6 to 8 years, who were beginning to learn to read. They aimed to examine the relationship between reading fluency (reading accuracy and speed), reading comprehension, and reading motivation. For this purpose, they differentiated three components of motivation: (i) reading self-concept or perceived self-competence, according to the terms proposed by Ryan and Deci (2000, 2020); (ii) value of reading, that is, having the belief that reading is useful and beneficial; and (iii) literacy aloud, linked to social interactions during story reading. The results showed a significant positive correlation between reading comprehension and each of the three components of reading motivation; however, reading fluency was correlated only with reading self-concept.

The nature of reading motivation is complex and multidimensional, and is affected by internal and environmental factors such as literacy in school and at home (Baker and Scher, 2002; Nevo et al., 2020; Nevo and Vaknin-Nusbaum, 2020; Altun et al., 2021; Cho et al., 2021). In particular, improving the perceived value of one’s own skills and goals depends on the combination of several elements, including the environment in which the child develops, and the practices carried out at home. Thus, motivation may be either inhibited or enhanced by parental expectations and the importance they attach to the specific behavior, task, or domain (Baker and Scher, 2002; Ruiz et al., 2019). In other words, parents who enjoy reading and hold it in high regard will be concerned about their child’s reading performance and will foster experiences that encourage reading at home and a reading routine. These practices and experiences would also include literacy aloud moments, for example, through storytelling or shared reading.

#### 1.2.4. Interaction between home literacy environment, motivation, and reading

Parents’ expectations that lead them to carry out certain activities may constitute the central axis of an active HLE, made possible by the power of affective relationships between parents and children and the motivating capacity of parents, as well as by the availability of families to dedicate time and attention to these practices (Sonnenschein and Munsterman, 2002). Adults create spaces for leisure and enjoyment with the children around literacy activities. This makes children want to repeat the activity and learn while performing it, not so much because of the activity itself but because of the agents involved in it. As time passes, their performance on literacy tasks improves, and consequently, their reading self-concept and the value they place on reading increase, and they develop an interest in it. It is noteworthy that, at an early age, the value of reading depends on the rewards received by the child in the short term, and the degree of usefulness and benefit of reading perceived by the child varies according to these rewards. However, as their reading experience increases and they approach adulthood, their assessment of the reading actor changes, anticipating or expecting medium and long-term benefits, in addition to immediate internal rewards (enjoyment of the narrative, distraction and escape from other problems, acquiring new knowledge on a topic of interest, etc.).

The child establishes a reading routine guided by extrinsic motivation, the main reinforcer being the leisure time with family performing a prolecting or reading task. Together, these points suggest that families begin the process of scaffolding reading in the child’s early years by creating an HLE. This HLE evolves and transforms based on modifications in the role played by the agents that compose it and the resources they possess, adapting to the child’s zone of proximal development at each time point. Meanwhile, the active HLE is maintained due to the fact that it manages to enhance the main motivational aspects involved in reading in the early years of learning to read and schooling. In particular, emotional reactions and feelings are enhanced through the social interactions and positive interpersonal relationships that are established and developed, based mainly on affection and pleasant experiences, giving rise to a feeling of belonging and connectedness inherent to building a relationship. In turn, there is an improvement in children’s reading performance, and consequently, in their reading self-concept or need for competence, which benefits from structured environments with positive feedback and learning facilitators (Deci and Ryan, 2000; Ryan and Deci, 2017, 2020). In such an environment, adults begin by acting as a role model, for example, by reading aloud stories to their children. As the child’s linguistic and cognitive development progresses, the activity varies such that the child takes an increasingly active role in the task by modifying the activities (reading together, reading aloud by the child to the adult, internal reading by the child, etc.), under parental guidance and supervision. Parents continue to gradually eliminate direct supervision of the task and focus on stimulating the activity indirectly—for example, through conversations about books—until the child is able to read autonomously. However, it should be noted that, in order to achieve the ultimate goal of reading independently and frequently, the causal attributions that the child establishes for his or her successes and failures during reading as well as the value of the goal achieved are essential, among other factors, thanks to the beliefs and expectations transmitted by the family throughout the child’s development.

Baker et al. (2001), Bus et al. (2000), and Sonnenschein and Munsterman (2002), among others, collected measures of verbal interactions and the nature of those interactions during literacy activities such as shared reading as an indicator of affective appraisals. Baker et al. (2001) confirmed that conversations associated with the meaning of the text between parents and children—in the first years of schooling—were correlated with more positive affective appraisals, compared with conversations about the process of identifying the written code (reading decoding). Whereas, Sonnenschein and Munsterman (2002) investigated the influence of the affective quality of interactions between parents and children, aged 5 to 6 years, during reading and the type of discourse produced in these interactions on components of literacy (i.e., phonological awareness, print orientation, story comprehension, and children’s motivation for reading). Their results demonstrated that students who experienced more positive reading interactions at an early age showed greater motivation for reading in the first grade (between 6 and 7 years of age).

The types of interaction between adults and children during literacy activities have also been studied by Luo and Tamis-LeMonda (2017) and Patel et al. (2021) who identified differences between interactions based on “immediate” questions (related to immediate memory and literal comprehension of the narrative) and “non-immediate” questions (associated with developing hypotheses and making inferences, comparing stories, comparing fiction with reality, etc.). They found that: (i) children show reciprocity and participate actively in conversations; (ii) children tend to respond with questions similar to those of their parents, whether “immediate” or “non-immediate,” of lower or higher level of difficulty, thus imitating the type of interaction; and (iii) parents increase their interactions when they perceive greater involvement on the part of their children.

The active HLE model involves even greater interest and availability on the part of families, which are directly dependent on parents’ expectations and perceived value of the task. As stated by Baker and Scher (2002), a positive attitude on the part of adults, including beliefs about the importance and pleasure of reading, influences their children’s enjoyment of reading. Thus, the more knowledge adults have about the benefits of reading, the greater their concern for their children to read correctly and the greater their interest in designing a literate environment.

Wiescholek et al. (2018) explored the perceptions of a group of 281 children aged 6 years and their parents regarding passive and active HLE. Specifically, they investigated the relationship between the children’s perception of HLE and their parents’ educational level, enjoyment of literacy, reading frequency, and phonological awareness. The adults completed a questionnaire assessing their educational level, while the children completed a questionnaire with questions about the number of books in the home (an indicator of passive HLE), and another five questions related to the frequency of literacy activities performed in the family (active HLE). The frequency and enjoyment of reading were assessed using the German version of the Children’s Interest Measure scale (Baroody and Diamond, 2012), while the standardized PB-LRS test (Barth and Gomm, 2008) was used to collect measures of phonological awareness. On analyzing the data, among other findings, the researchers observed that a passive HLE was more prevalent in households where parents had a higher level of education, and that both dimensions were associated with children’s enjoyment of reading. However, only active HLE was associated with a higher reading frequency. This leads us to suppose that, in children who are advanced in their linguistic and reading development (with an adequate phonological level, who have mastered grapheme-phoneme conversion strategies and overcome their difficulties in reading recognition, etc.), the implementation of an HLE is not sufficient. It would not be enough to implement a passive HLE program to increase the taste and enjoyment of reading, or to establish a reading habit that lasts over time and in adulthood; it would be more beneficial to implement an active HLE that also seeks to improve other variables and processes of greater complexity (readers, motivational …).

Meanwhile, several researchers allude to the influence of parents’ positive beliefs about their ability to assist their children in acquiring skills and learning to read on children’s reading motivation and emergent literacy skills. They suggest that parents’ positive beliefs about literacy are directly related to the quality of literacy activities carried out at home (Bingham, 2007; McElvany and Van Steensel, 2009; Bojczyk et al., 2016; Patel et al., 2021).

Therefore, we propose to reformulate the existing concept of an active HLE, based on the understanding that it not only improves the psycholinguistic and cognitive variables traditionally studied (phonological skills, reading fluency or comprehension), but also enhances motivational aspects associated with reading in children, including components such as self-concept or reading competence, sentimental and affective relationships, parents’ expectations and motivation to improve their children’s reading, and the home literacy environment.

Consequently, this study pursued the following objectives. First, it aimed to confirm families’ expectations about the future influence of an active HLE program on the improvement of positive affective relationships between parents and children, reading performance (reading speed and comprehension), and children’s motivation to read, before starting the intervention at the first evaluation time point. In line with this, the second objective was established, seeking to confirm teachers’ expectations about the future effects of active HLE training on the abovementioned variables at the same evaluation time point, allowing us to reflect on whether the expectations of both groups coincide and on the effects of the HLE on the children.

Once the expectations of the two groups of adults had been verified, the active HLE program was started and teachers and families were evaluated at three different time points, with the aim of improving the expectations of both groups regarding the influence of the program on positive affective relationships between parents and children, reading performance (reading speed and comprehension), and children’s reading motivation after 18 months of training (two academic years). However, prior to the end of the program and this last evaluation, measurements of all variables were collected twice—after 9 months of intervention and 3 months without training (due to summer vacations)—to monitor and control the study variables.

In summary, firstly, we hope to verify that both families and teachers hold positive expectations regarding the future influence of an active HLE program on improving positive affective relationships between parents and children, reading performance (reading speed and comprehension), and children’s motivation to read, prior to commencing the intervention or the initial assessment. A motivational aspect highly linked to the interest, quality, and frequency of educational and reading practices carried out by adults. Therefore, if these expectations are high, we would anticipate increased interest and involvement in the HLE program – from the outset of the intervention – on the part of teachers and family members, requiring fewer training sessions.

Secondly, we expect to enhance the expectations of both groups of adults—families and teachers—concerning the program’s impact on positive affective relationships between parents and children, reading performance (reading speed and comprehension), and children’s motivation to read, after 18 months of training (two academic years). Consequently, this improvement is expected to positively influence motivational aspects associated with the child’s motivation to read.

## 2. Materials and methods

### 2.1. Participants

To select the participants for this study, a cluster sampling method was employed involving parents and teachers of students aged between 6 and 8 years from a private school in Malaga, Spain. Families and teachers of two groups of students who were starting formal education in the Primary Education stage were included in the study. They implemented the active HLE program in their homes and monitored its progress in the classroom over two academic years (1st and 2nd grades of Primary Education). The selected school was located in a socioeconomically and culturally middle-to-high-class area, which allowed controlling for other variables that might influence the program’s implementation and outcomes, such as the families’ linguistic and reading levels, educational background, etc.

It is worth mentioning that the school’s management team, teaching staff, and families were interested in incorporating the active AFA model as the school’s reading program. Considering the school and families’ preferences, no family members or teachers within the specified age range were excluded from the study.

The adults were divided into two groups. One group consisted of 54 family members with an average age of 43 years, which included 85% of participants with a university education and 75% of individuals who were already working. The other group comprised two teachers in charge of tutoring their students, with an average age of 55 years, and 30 years of experience as teachers and classroom tutors in their current school (see Table 1).

**Table 1:** Groups and characteristics of the study participants.

Groups of participants	N	Average age	Percentage of participants with university studies	Percentage of participants who have joined the workforce
Family members	54	43	85%	75%
			**Average years of professional experience**	
Teachers	2	55	30 ages	

The following inclusion criteria were established for the selection of participants. On the one hand, with respect to families:

- The child should have been in the 1st grade of primary school at the beginning of the program.
- The children must have attended the same school, a charter school in the city of Malaga.
- The child could not receive, or have received, specific reading acceleration programs.

On the other hand, for teachers, the following inclusion criteria were determined:

- Acting as a tutor for the group of students participating in the project.
- Maintain their permanence in the study, as a tutor for the same group of students, until the end of the project.

The study was approved by the Ethics Committee of the University of Malaga (145-2021-H).

### 2.2. Instruments

Four instruments, two of which were aimed at families and the other two at teachers, were used to measure the variables in this study (that is, family and teacher expectations regarding the influence of an active HLE program—before and after its application—on positive affective relationships between parents and children, children’s reading ability, and reading motivation). All instruments were designed *ad hoc* using Likert-type scales with five response options. Depending on their level of agreement, participants had to check one of the boxes from 1 to 5 (1 being “totally disagree” and 5 “totally agree”).

Regarding the composition and selection of items for the scales, the collaboration of five expert judges in the field was sought. All of them had extensive research experience in the disciplines of psychology, educational psychology, and education, as well as clinical and/or teaching practice. These judges evaluated each item based on its relevance, suitability, and comprehensibility, resulting in a high level of agreement (Cohen’s Kappa 0.9) among all of them. This process led to the creation of a scale with high interjudge reliability (Cohen et al., 2011; León and Montero, 2015).

1. *Scales on expectations about the future active Home Literacy Environment program and its effects on children.* These scales were used prior to the implementation of the program. Two independent scales, one aimed at families and the other at teachers, were used to measure expectations about the influence of the future active HLE program on the improvement of affective relationships between parents and children, children’s reading ability (comprehension and speed), and reading motivation. In relation to the scale aimed at family members, 5 items were selected from a total of 10 items that made up the scale; whereas, in the scale aimed at teachers, 6 items were included from the total 17 (see Appendix A).
2. *Scales on expectations about the active Home Literacy Environment program and its effects on children.* These scales were used during and after the implementation of the intervention program. Two independent scales, one aimed at families and the other at teachers, were used to measure expectations about the influence of the active HLE program on the improvement of affective relationships between parents and children, children’s reading ability (comprehension and speed), and reading motivation. In relation to the scale aimed at family members, 5 items were selected from a total of 10 items that made up the scale, while in the scale aimed at teachers, 6 items were included from the 17 that made up the scale (see Appendix B).

### 2.3. Procedure

**Figure 1:**
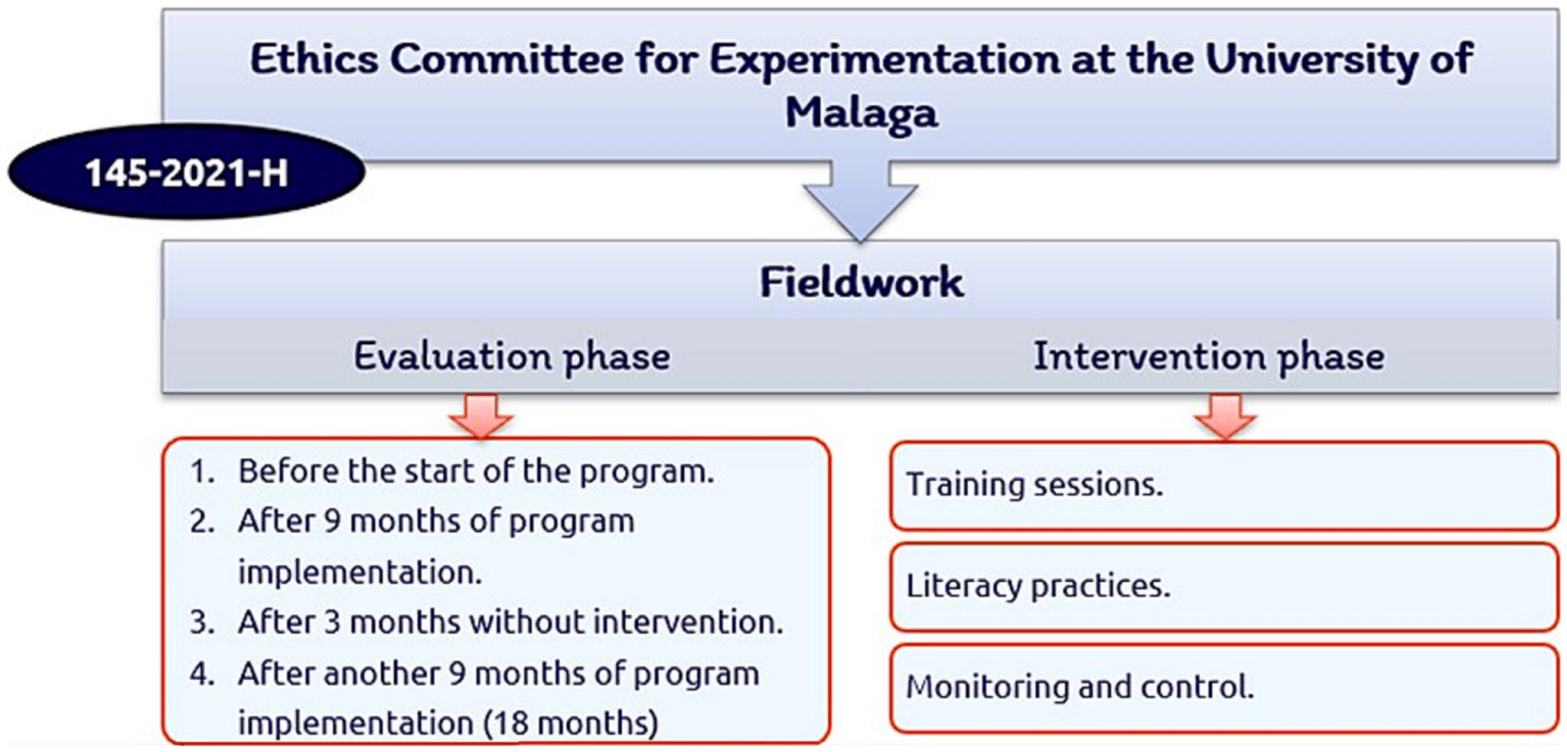
Phases of the work procedure.

The first step in the project was to select the participants. Subsequently, the informed consent form and pertinent authorizations were signed, and field work was started (see Figure 1).

#### 2.3.1. Evaluation phase

The variables were evaluated at four different time points, that is, at the beginning and end of two school years: (i) before the application of the program, (ii) after 9 months of intervention, (iii) after 3 months of summer vacation, during which no training was provided, and (iv) after another 9 months of application of the active HLE program.

#### 2.3.2. Intervention phase

The intervention began with counseling for families and teachers, explaining what the program consisted of and the benefits of an active HLE. Then, they were provided with the necessary tools for the implementation of the intervention. To this end, two training sessions were held for families and two for teachers.

The main literacy task, around which the program revolved, was reading aloud by the child to the adult, four times a week for 10–15 min. The person in charge of reading with the child had to correct the reading decoding errors detected, in addition to promoting reading comprehension (activation of previous knowledge, short-term memory…) through four questions related to the text (for example, who were the characters in the story, why they could act or feel the way they did, etc.). Families were also encouraged to enhance their enjoyment of reading through comments and conversations with their children about the books read, comparison of characters, and so on.

**Figure 2:**
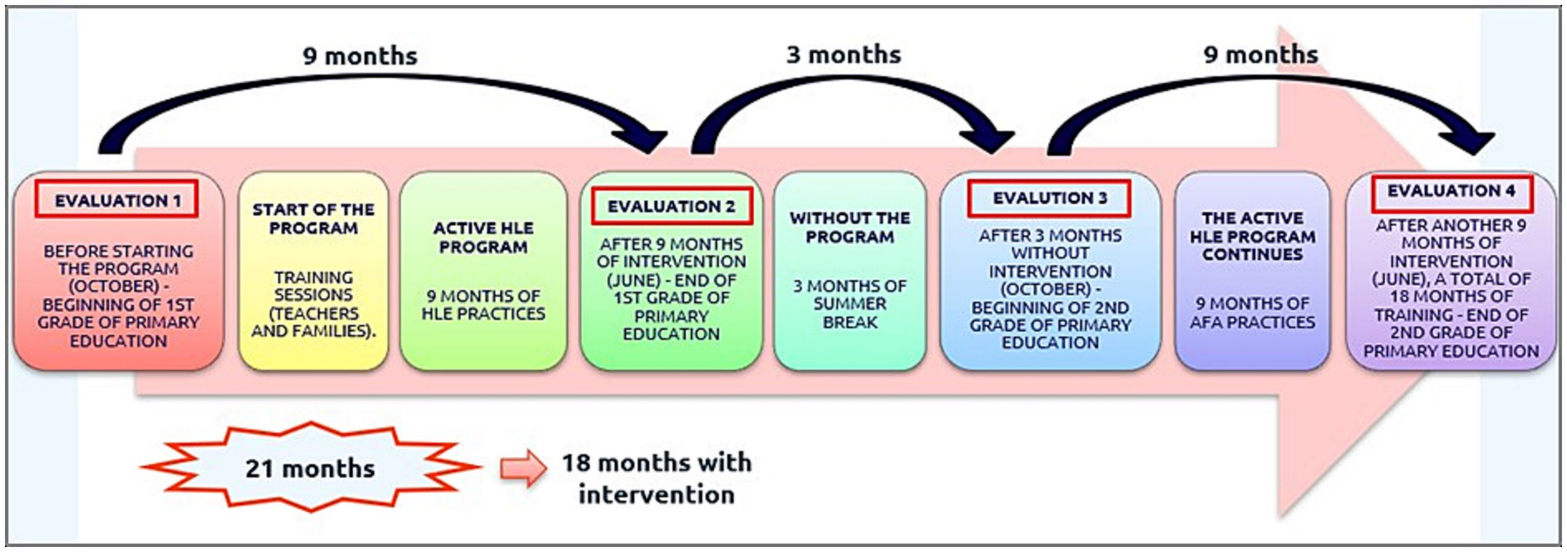
Schedule for the implementation of active HLE.

To ensure that the reading was appropriate to the linguistic and reading level of the children and to include different themes, reading material—narrative texts—was purchased according to the level of curricular competence of the children. The teachers chose 54 books, which were exchanged every Friday. The teachers dedicated 1 h to the book exchange, ensuring that each child had a different book every week to perform the literacy activity at home. In this way, the teachers not only had the opportunity to exchange reading material but also to encourage students’ interest in the program and, more importantly, in reading, through conversations with the students about the texts, their opinions and tastes, and even those of their families (see Figure 2).

**Figure 3:**
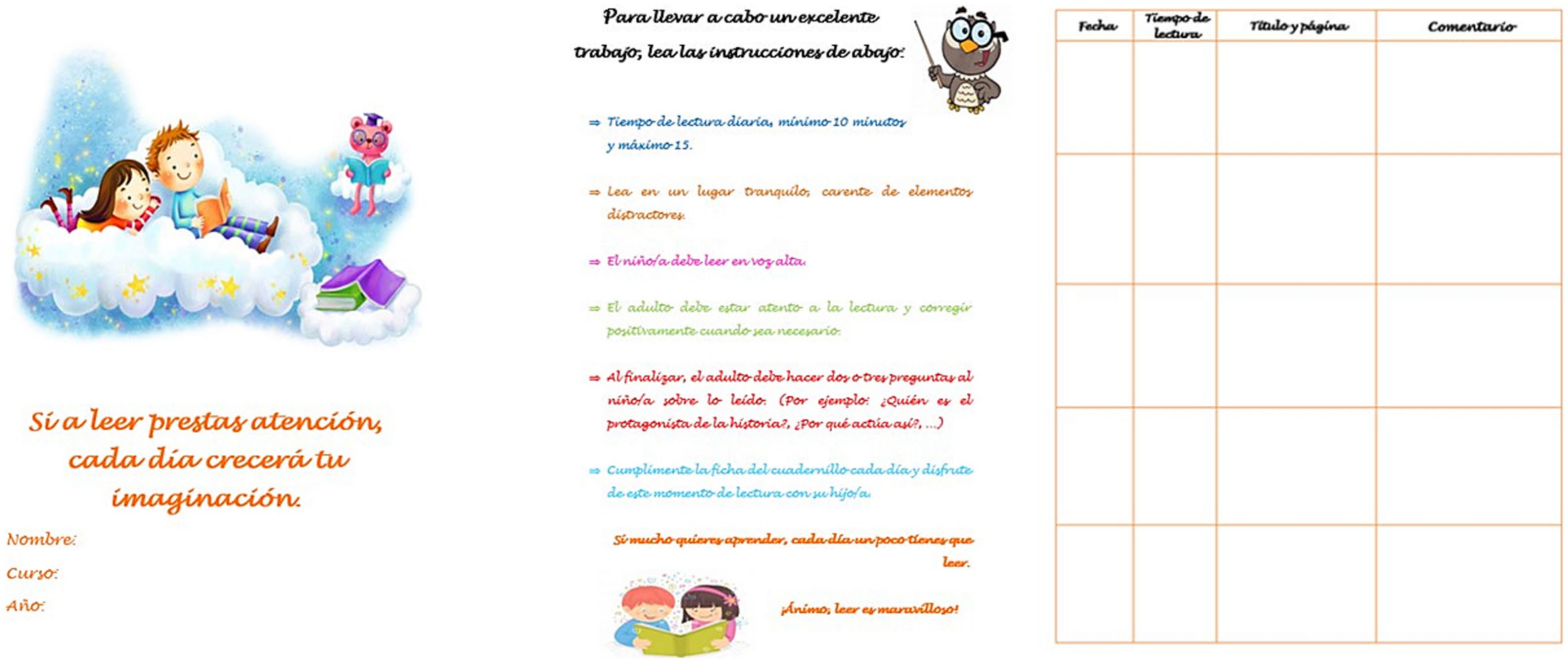
Daily reading log.

To monitor the intervention, a notebook or daily log was designed to record data such as the date, title of the book, pages read, difficulties encountered, doubts, praise, and so on (see Figure 3). These notebooks were reviewed daily by the teachers and weekly by the research team. In addition, a brief summary was included with the instructions and fundamental indications for the application of the active HLE program, previously explained in the training sessions (see Figure 3).

### 2.4. Data design and analysis

The present study proposed the application of the quantitative method and two different designs (Cohen et al., 2011; León and Montero, 2015). The first was a quasi-experimental pre-post design with a single group, with the objective of establishing a relationship between the independent variable (active HLE program) and the dependent variables (parents’ expectations about the influence of the HLE program on the improvement of positive affective relationships between parents and children, children’s reading performance, and children’s reading motivation). Second, a single-case ABA design was adopted to identify the relationships between the independent and dependent variables (teachers’ expectations about the influence of the HLE program on the improvement of positive affective relationships between parents and children, reading performance, and reading motivation of their students).

Statistical analyses were performed using Statgraphics 18 and SPSS 24 programs, beginning with an analysis of the descriptive statistics of each variable. To compare the scores obtained for all variables at the four different evaluations, a simple analysis of variance (ANOVA) with repeated measures was performed. Finally, to detect improvements in the variables, significant differences were observed in each factor.

## 3. Results

First, to confirm families’ expectations regarding the influence of an active HLE on positive affective relationships between parents and children, children’s reading performance (reading speed and comprehension), and motivation to read, a descriptive analysis of all the variables was performed at the first evaluation, prior to the implementation of the intervention program (see Table 2). The data showed that, from the beginning of the study, the families presented a positive and high evaluation of the effect of an active HLE on affective relationships between parents and children (M = 3.81), their children’s reading performance (M = 8.59), and motivational aspects involved in the act of reading (M = 8.54).

**Table 2:** Summary statistics.

		Evaluations	Average	Standard deviation	Coefficient of variation	Minimum	Maximum	Range	Standardized Skewness	Standardized Kutorsis
Positive emotional relationships		1	3.81	1.05	27.56%	1	5	4	−1.6	−0.64
		2	4.26	0.83	19.44%	3	5	2	−1.5	−2.01
		3	3.83	1.09	28.55%	1	5	4	−3	1.01
		4	3.92	0.9	23.13%	1	5	2	−2.37	1.27
Reading ability	Reading comprehension	1	4.04	0.83	18.97%	2	5	3	−4.52	2.81
		2	4.57	0.72	15.66%	2	5	3	−5.12	3.77
		3	4.31	0.69	16.12%	3	5	2	−1.55	−1.19
		4	4.59	0.59	13.05%	3	5	2	−3.57	0.69
	Reading speed	1	4.55	0.74	16.33%	2	5	3	−5.73	5.55
		2	4.72	0.59	12.62%	2	5	3	−7.81	11.97
		3	4.57	0.66	14.47%	3	5	2	−3.89	0.73
		4	4.65	0.55	22.94%	3	5	2	−3.93	1.25
Motivation and enjoyment of reading	Time dedicated to reading homework in the family	1	4.02	0.94	23.42%	2	5	3	−1.8	−0.86
		2	4.13	0.91	22.08%	2	5	3	−1.72	−1.28
		3	3.76	1	26.81	1	5	4	−1.22	−0.55
		4	4.06	0.88	21.64%	2	5	3	−0.85	−1.86
	Conversation about reading	1	4.51	0.63	14%	3	5	2	−2.95	−0.08
		2	4.63	0.59	12.79%	3	5	2	-4.12	1.43
		3	4.52	0.66	14.73%	2	5	3	−4.39	3.95
		4	4.53	0.64	14.01%	3	5	2	−3.17	0.13

It is also interesting to note that, on comparing the results of teachers with those of families, the teachers presented lower expectations about the effects of an active HLE on the reading motivation (M = 7.5) of their students; however, after the implementation of the program, the teachers’ mean scores regarding the improvement of positive affective relationships between parents and children (M = 3.5) and children’s reading performance (M = 8.5) were similar to the families’ scores.

Second, to verify whether these expectations improved after the application of the active HLE program, descriptive analyses of all the selected variables were conducted on the following three evaluation occasions: evaluation 2, after 9 months of training; evaluation 3, after 3 months of vacation period (without intervention); and evaluation 4, after nine more months of application of the active HLE program. The results confirm that the positive expectations regarding the influence of an active HLE on the variables remained high at all four points in time, that is, from the beginning to the end of the active HLE intervention program. However, it should be noted that the observations made by the families were slightly asymmetric to the left, that is, they presented a standardized Kurtosis or negative Skewness statistic due to the low scores of a few individuals, possibly the most critical and demanding families, whose results did not evolve or behave similarly to the rest of the participants (see Table 1). These results, which were high at all four evaluation occasions, are also presented in the data obtained from the teachers, when compared with the average values found in parents’ responses to the questionnaires. Thus, the highest averages were observed in the fourth evaluation, increasing up to four points between the first and last evaluation, that is, two academic years and 18 months of training, for the reading motivation variable.

Finally, to measure the efficacy of the active HLE program for improvements in the variables, a simple repeated measures ANOVA was performed and the changes were observed at the four evaluation time points. Additionally, sphericity was tested with the Mauchly Test of Sphericity. Thus, after the first 9 months of training, significant differences (*value of p* = 0.0273) were detected in affective relationships between parents and children and children’s reading performance. That is, families perceived improvements in both these aspects after the first period of application of the active HLE program.

These improvements were maintained until the end in terms of the children’s reading performance as perceived by their parents; however, the same was not true for the remaining variables. Specifically, within the framework of reading performance, the families’ perception of their children’s reading speed and comprehension was included. After the descriptive and variance analyses, as there were no high correlations between the variables and there were only two indicators, a fact corroborated by an exploratory factor analysis (AFE), we decided to conduct a separate study. This allowed us to observe that the scores are high for all variables on all four occasions, but there was a phenomenon that was repeated, as reading speed had a descriptively higher average in the four measurements, obtaining significantly higher values at evaluations 2 and 3, that is, after the first academic year and 9 months of intervention (W = 0.005, *value of p* = 0.000). Whereas, significant differences in reading comprehension (*F* = 3.238, *value of p* = 0.026, η^2^ = 0.058) were found at evaluations 2 and 4, at the end of each academic year and the end of the active HLE intervention program (after each 9-month stretch of training).

With regard to expectations about reading motivation, it should be noted that, although no significant differences were found (*value of p* > 0.05) throughout the study period, high scores were obtained on all four evaluation occasions. Thus, the families started out with positive expectations regarding the influence of an active HLE on their children’s motivation and enjoyment of reading (M = 8.54; maximum score = 10), which were maintained after 9 months of training (M = 8.76), 3 months without any intervention (M = 8.28), and nine more months of application of the active HLE program (M = 8.59).

The reading motivation variable is composed of two items which, despite establishing significant correlations, were weak, a fact confirmed after AFE, which led to further study of the items separately. This analysis showed that the descriptive average of one of the items, which refers to the effect of time spent reading by children under parental supervision, on motivation for the activity is always slightly lower. However, the item with slightly higher scores focuses on the effect of holding conversations about reading as a family on children’s reading motivation. Consequently, the data suggest that, although not significant, parents perceive that their attitude toward reading, conversations about books, and their role as a reading role model would have a slightly greater influence on their children’s motivation than the amount of time spent reading.

In summary, prior to the application of the active HLE program, families and teachers had high and positive expectations of the effects of the program on affective relationships between children and parents, children’s reading performance, and reading motivation. These expectations were maintained throughout the study, and, in the case of family members, there were significant changes and improvements—after the first 9-month training period—in the quality of affective relationships and reading performance of their children. At the end of the study, significant improvements were maintained in reading performance.

## 4. Discussion

This study, framed within a larger project, was conducted to explore the expectations of families and teachers of a group of 54 students aged 6 to 8 years, regarding the effects of HLE on positive affective relationships between parents and children, children’s reading performance (reading speed and comprehension), and reading motivation, before and after the implementation of an active HLE program.

### 4.1. Main findings and the implications of these results

In relation to the first objective, the results showed that the parents and teachers had high expectations regarding the effects of the HLE program on all measured variables from first evaluation occasion before the implementation of the program, that is, from the beginning of the project. These high expectations, far from being a drawback, are very important and valuable, as it is desirable for all participating families to share these values. These findings are in line with those reported by authors such as Baker and Scher (2002), and Ruiz et al. (2019), who emphasized the influence of parents’ positive attitudes and beliefs toward reading on their children’s motivation and pleasure in reading, through the promotion of literacy practices. More importantly, these positive beliefs are also fundamental to the design of quality literacy activities (Bojczyk et al., 2016; Patel et al., 2021). Based on these, the involvement of the families in this study may have been greater when considering that the act of reading and HLE have a high influence on their children, beginning their incursion into the active HLE program due to the motivating effect of their expectations, which, taking into account the scores of the teachers in charge of tutoring the students in the Educational Center would be enhanced by the attitudes of the teachers and the initial training sessions.

The second objective was to improve and maintain the expectations of families and teachers regarding the effects of the active HLE program on positive affective relationships between parents and children, children’s reading performance (reading speed and comprehension), and reading motivation after the application of the intervention for 18 months. In contrast to the results obtained by Wiescholek et al. (2018), no significant differences were found in the students’ reading motivation. Regarding this variable, it is worth noting that motivation for reading is a complex psychological construct influenced by numerous factors and aspects that vary and evolve over time and with increasing task demands. This fact, along with the results obtained, leads us to believe that designing instruments to measure motivation in young children, regarding the active HLE model and reading, is equally complex and requires a thorough evaluation of each of its components. As observed throughout the study, despite not perceiving changes in their children’s motivation for reading, parents do recognize improvements in motivational aspects that contribute to their interest in reading. Two other factors in this study that could be influencing the results are: on the one hand, the effects of an active HLE on students’ reading motivation were not directly assessed through the child, but data were collected on the effects of different active HLE practices on children’s reading motivation as perceived by families and teachers; on the other hand, and even more important, the statistical averages extracted in this study were high from the beginning of the project until its completion, thus allowing a smaller margin for improvement. It is also interesting to note that the aspects perceived by the families that most influence reading motivation were the conversations around reading, rather than the time the child spent reading aloud supervised by the parent. In other words, adults attached slightly greater importance to their role as role models, their attitudes, and the time spent together after reading, in which they conducted dialog about the content of the reading material; the adult asked a series of questions to enhance children’s reading comprehension and exchanged opinions and impressions. This suggests that this space of time dedicated to conversations about reading also coincides with one of the most important occasions for fostering affective relationships between parents and children, creating positive experiences together.

In line with this, Baker et al. (2001) showed that conversations associated with the meaning of the text between children and parents are correlated with more positive affective evaluations than conversations about reader recognition. This aspect should be taken into account when interpreting another finding of this study—the significant improvements obtained in the expectations of the effect of the active HLE program on positive affective relationships between parents and children, after 9 months of intervention. As indicated by Sonnenschein and Munsterman (2002), the affective quality of the interactions between parents and children aged 5–6 years during reading influences reading motivation when the children reach the first grade (between 6 and 7 years of age). Consequently, although the participants did not consider that the HLE and the program significantly improved their children’s interest and enjoyment of reading, they maintained their high positive expectations about its influence on positive affective relationships at home, and therefore, on one of the fundamental components of both HLE and reading motivation. These interactions arising from the affective family bond would produce emotional and sentimental reactions that are fundamental for the promotion of the taste and enjoyment of reading, as well as for the implementation and maintenance of an active HLE, constituting a motivational aspect to be taken into account in this study, which should be investigated independently and in relation to the reading motivation variable.

Finally, significant improvements in students’ reading performance, as perceived by families, were also observed after the implementation of the full active HLE program (lasting two academic years). According to the data obtained by other researchers such as Niklas et al. (2020) and Niklas and Schneider (2014, 2017); among others, as well as those found in two studies that make up our research project, one of which has been published (Romero-González et al., 2021). Specifically, families detected a significant improvement in reading speed at evaluations 2 and 3 (after 9 months of training and 3 months of vacation without intervention), a phase of learning in which children were automating the process of reading recognition, as well as in reading comprehension at evaluations 3 and 4 (at the beginning and end of the second academic year, after the application of the entire program), when fluency errors had been reduced, children had advanced in their general psycholinguistic development (phonological awareness, vocabulary, oral comprehension, etc.), and could use more cognitive effort in text comprehension. Based on existing literature, although it is not part of the variables analyzed in this study, we believe that the effects of an active HLE on children’s reading ability as perceived by families should lead to benefits in their learning and, above all, in their interest in learning, particularly learning to read (Baker and Scher, 2002; Wirth et al., 2020). This would be explained by the fact that students would show an increase in their level of perceived competence during the task, acquiring and developing confidence in the resolution of reading activities and improving their reading self-concept, another motivational components of great relevance for interest in reading (Nevo et al., 2020; Nevo and Vaknin-Nusbaum, 2020).

In summary, the adults began the active HLE program with high expectations about its future effects on the different variables, which were maintained after its implementation. This is important, because it allows the application of the HLE program in better conditions, facilitating its implementation as there is no need to implement specific improvement aspects of expectations. In addition, it can be affirmed, thanks to the training families improved their expectations about the influence of the active HLE on fundamental motivational components for reading, such as positive affective relationships at home and children’s reading performance, on different evaluation occasions. The improvements observed in the parents’ perception of children’s reading ability lasted until the end of the 18-month active HLE program; taking into account the results of previous studies, this indicates an increase in perceived reading self-competence, and therefore, in the child’s reading self-concept, which is fundamental for the child’s taste and enjoyment of reading.

### 4.2. Limitations and future lines of research

Some limitations were encountered during the research, which should be noted. The first was the impossibility of including a control group for ethical reasons, as the families and the teaching staff of the educational center requested that all the children in the class receive the training. It was not possible to include a group of participants, that is, families and teachers, with children who did not participate in the active HLE program, with the same characteristics and from the same context as those who completed the 18-month intervention.

Second, to control for variables such as cultural and linguistic differences, the reading level of the families, their accessibility and availability for work reasons, and so on, we chose to conduct the active HLE program with children from a middle and upper middle class charter school in the city of Malaga, and their respective families and teachers. However, in the future it would be interesting to carry out applied research with larger and more heterogeneous samples of participants, and examine socioeconomic and cultural variables, including linguistic and reading levels. This would allow generalizing the results to other contexts, different adults, and children with diverse personal and family characteristics, often in more disadvantaged settings and less enriched environments.

## 5. Conclusion

Research on HLE formally began at the end of the 20th century, defining this term as the quantity and quality of resources and skills that families possess to design environments that facilitate reading and learning to read for young children. HLE highlights the role of the family as a model and reinforcer during children’s learning in general, and reading, in particular.

Studies on HLE have increased in the past few years, mostly focusing on the influence of HLE—passive and/or active—on the language and reading ability of children aged approximately 4 to 6 years (Sénéchal and Young, 2012; Silinskas et al., 2012; Niklas and Schneider, 2014, 2017; Boerma et al., 2017; Dong et al., 2020; Korucu et al., 2020; Niklas et al., 2020; Sénéchal, 2020; among others). However, due to the complexity of these studies and the numerous variables that need to be controlled, applied research conducted with older children—aged 6 to 8 years—focusing on the active dimension of HLE is less frequent. This includes research on the attitudes and role model behavior of families, as well as the types and characteristics of reading and reading practices and activities carried out at home.

In this study, framed within a larger project, we pursued and managed to deepen all these aspects, and further, analyzed the effects of an active HLE on variables other those traditionally investigated; we included variables that are involved in reading and the acquisition of a reading routine, such as motivational aspects and the influence of affective bonds between parents and children during literacy. To this end, following previous studies (motivation, attitude in activities…) and taking into account the multifactorial character of HLE, a possible cause of the continuous emergence of new classifications, dimensions, and types of literacy practices, we suggest a novel classification of literacy practices (Burgess, 2002, 2011; Sénéchal and LeFevre, 2002, 2014.). We provide a redefinition of the concept of active AFL, which was the basis of the active HLE program designed in this study.

Children become involved in reading activities with their families motivated by the leisure time shared with adults with whom they share a strong affective bond, and not so much by the task itself. These approaches to reading allow children to train their reading ability and enjoy it, which raises their level of perceived competence and increases their achievement motivation, leading to internal, stable, and controllable attributions for their reading successes, resulting in an improvement in motivation and reading frequency and in autonomy at older ages. Most importantly, just as this type of environment is possible thanks to the positive affective relationships established between parents and children, the latter are nurtured and benefit from it (generating moments that allow parents and children to continue getting to know each other, have common views and tastes, etc.). As a consequence, these types of relationships remain in the memory linked to a positive valuation of the time and moment lived, as well as of the activity around which these spaces of enjoyment revolve; an active HLE provides a context that favors the creation of opportunities to establish and strengthen these affective bonds.

According to the first and second objectives of the study, the obtained results confirm that both families and teachers’ expectations regarding the future influence of the active HLE model on affective relationships between parents and children, the reading ability of young children, and their motivation to read, are high from the beginning before the intervention program’s implementation. As for the third objective, it is verified that these family expectations remain throughout the entire study, from the start of the intervention program until its completion. Moreover, among the various variables included in this study, families consider that the variables that have benefited the most from the active HLE intervention are affective relationships—specifically, the conversations held after reading tasks—and reading performance. Both components are fundamental for reading motivation and are therefore essential aspects for the development of the active HLE model.

Regarding the fourth and final objective, it is worth noting that the high expectations also persist among teachers, who highlight changes in children’s behavior during recess. The children started including discussions and opinions about books, comparisons, evaluations, and recommendations to their peers during playground conversations.

These data, together with previous findings, induce us to reflect on the role of the teacher, because it should not be forgotten that teachers’ perform constant and weekly supervision of the active HLE program. Therefore, although not directly measured in this study, their high expectations and involvement in the project suggest that their role could be more relevant than expected. Teachers have several functions beyond training their students, including providing positive reinforcement and collaborating in the design of a facilitative context for learning, directly influencing the children’s motivation (Ryan and Deci, 2020). Moreover, through tutoring, they train and advise students’ families, transmitting knowledge and the importance and benefits of literacy practices, as well as providing feedback and support to the families. The tutoring program also offers feedback and reinforces the families’ successes. In short, we believe that this could not only impact the young children’s motivation, but also that of the adults in charge of implementing and guiding the training, and could emerge as a key component in active HLE.

These findings indicate new lines of research. Just as an active HLE should be adapted to the child’s psycholinguistic and reading development and level, modifying the type of task as the child advances in age and maturity, it could be interesting to design active HLE programs in which the central task revolves around such conversations about reading. In populations similar to our participants’ children and students, from the age of eight onwards, most children are able to read autonomously and with less supervision. Without requiring training for the improvement of phonological skills or reading decoding, it may be more interesting to promote deep reading and the motivational aspects associated with it. Thus, in the scaffolding that is built through an active HLE, parents must withdraw and transform their guidance to adapt to the new zones of proximal development, focusing their efforts on increasing children’s motivation for deep reading, with the ultimate goal that they read autonomously and enjoy it.

Finally, although there is a need for further research on the subject and for applied research to shed light on its implications in populations with different characteristics, we believe that this study provides new data that could be useful to the scientific and educational community. On the one hand, the expectations of parents and teachers in this study corroborate the benefits of active HLE on the improvement of psycholinguistic, cognitive, and reading processes and skills in young children, which have been directly evaluated in previous studies and showed similar results (Romero-González et al., 2021). On the other hand, the possible relevance of motivational and affective aspects is confirmed, not only for the acquisition of a habit and taste for reading, but also for the development and success of an active HLE.

## Data availability statement

The raw data supporting the conclusions of this article will be made available by the authors, without undue reservation.

## Ethics statement

The studies involving humans were approved by Comité Ético De Experimentación De La Universidad De Málaga (CEUMA). The studies were conducted in accordance with the local legislation and institutional requirements. The participants provided their written informed consent to participate in this study. Written informed consent was obtained from the individual(s) for the publication of any potentially identifiable images or data included in this article.

## Author contributions

MR-G: Writing – original draft, Writing – review & editing. RL-C: Writing – original draft, Writing – review & editing. SG-T: Writing – original draft. GR-I: Writing – original draft. RJ-R: Writing – original draft. JR-P: Writing – original draft, Writing – review & editing.

## Funding

The author(s) declare that no financial support was received for the research, authorship, and/or publication of this article.

## Conflict of interest

The authors declare that the research was conducted in the absence of any commercial or financial relationships that could be construed as a potential conflict of interest.

## Publisher’s note

All claims expressed in this article are solely those of the authors and do not necessarily represent those of their affiliated organizations, or those of the publisher, the editors and the reviewers. Any product that may be evaluated in this article, or claim that may be made by its manufacturer, is not guaranteed or endorsed by the publisher.
