# Supplementary material for: Active Home Literacy Environment: parents’ and teachers’ expectations of its influence on affective relationships at home, reading performance, and reading motivation in children aged 6 to 8 years
Source: Front Psychol. 2023 Sep 22;14:1261662. doi: 10.3389/fpsyg.2023.1261662 (PMC10557458; doi:10.3389/fpsyg.2023.1261662)
Supplement: Supplementary file 1 [file Data_Sheet_1.docx]

Supplementary Material

# Appendix - Scales on expectations about the active Home Literacy Environment program and its effects on children

**Appendix A -** Scales on expectations about the future active Home Literacy Environment program and its effects on children

| **Items for teachers** | 1 item  Do you think that reading aloud daily by your students to their families will improve the relationship between your students and their parents? | 1 item  Do you think that the implementation of the active HLE programme, in which you increase the time spent reading by your students, under the supervision of their family members, will improve the students’ reading speed? | 1 item  Do you think that the implementation of the active HLE programme, in which you increase the time spent reading by your students, under the supervision of their family members, will improve students' oral language comprehension? | 3 items  Do you think that the implementation of the active HLE programme, in which you increase the time dedicated to reading by your students, under the supervision of their families, will improve your students' motivation to read?  Do you think that the application of the active HLE programme has made the books read a topic of interest among the group of students?  Do you think that discussing what they have read as a family encourages your students' interest in reading? |
| --- | --- | --- | --- | --- |
| **Items directed to the family** | 1 item  Do you think that reading aloud daily will improve the relationship between you and your child? | 1 item  Do you think that dedicating time to reading for your child, under your supervision, will improve his/her reading speed? | 1 item  Do you think that dedicating time to reading by your child, under your supervision, will improve his/her reading comprehension level? | 2 items  Do you think that dedicating time to reading for your child, under your supervision, will improve his/her motivation to read?  Do you think that discussing what you read as a family encourages your child's interest in reading? |
|  | **Affective relationships between parents and children** | **Reading speed** | **Reading comprehension** | **Motivation for reading** |
|  |  | **Reading ability** | |  |

**Appendix B:** Scales on expectations about the active Home Literacy Environment program and its effects on children

| **Items for teachers** | 1 item  Do you think that reading aloud daily by your students to their families has improved the relationship between your students and their parents? | 1 item  Do you think that the implementation of the active HLE programme, in which you increase the time dedicated to reading by your students, under the supervision of their families, has improved the students' reading speed? | 1 item  Do you think that the implementation of the active HLE programme, in which you increase the time dedicated to reading by your students, under the supervision of their family members, has improved the students' oral language comprehension? | 3 items  Do you think that the implementation of the active HLE programme, in which you increase the time dedicated to reading by your students, under the supervision of their families, has improved your students' motivation to read?  Do you think that the application of the active HLE programme has made the books read a topic of interest among the group of students?  Do you think that discussing the things they read as a family encourages your students' interest in reading? |
| --- | --- | --- | --- | --- |
| **Items directed to the family** | 1 item  Do you think that reading aloud daily has improved the relationship between you and your child? | 1 item  Do you think that the time spent reading by your child, under your supervision, has improved his/her reading speed? | 1 item  Do you think that the time spent reading by your child, under your supervision, has improved his/her reading comprehension level? | 2 items  Do you think that the time spent reading by your child, under your supervision, has increased his/her motivation to read?  Do you think that discussing the things you read as a family encourages your child's interest in reading? |
|  | **Affective relationships between parents and children** | **Reading speed** | **Reading comprehension** | **Motivation for reading** |
|  |  | **Reading ability** | |  |
